# *Arabidopsis* Myosin XI-K Localizes to the Motile Endomembrane Vesicles Associated with F-actin

**DOI:** 10.3389/fpls.2012.00184

**Published:** 2012-09-03

**Authors:** Valera V. Peremyslov, Amy L. Klocko, John E. Fowler, Valerian V. Dolja

**Affiliations:** ^1^Department of Botany and Plant Pathology, Center for Genome Research and Biocomputing, Oregon State UniversityCorvallis, OR, USA

**Keywords:** *Arabidopsis*, myosin XI, filamentous actin, endomembrane vesicles, transport vesicles

## Abstract

Plant myosins XI were implicated in cell growth, F-actin organization, and organelle transport, with myosin XI-K being a critical contributor to each of these processes. However, subcellular localization of myosins and the identity of their principal cargoes remain poorly understood. Here, we generated a functionally competent, fluorescent protein-tagged, myosin XI-K, and investigated its spatial distribution within *Arabidopsis* cells. This myosin was found to associate primarily not with larger organelles (e.g., Golgi) as was broadly assumed, but with endomembrane vesicles trafficking along F-actin. Subcellular localization and fractionation experiments indicated that the nature of myosin-associated vesicles is organ- and cell type-specific. In leaves, a large proportion of these vesicles aligned and co-fractionated with a motile endoplasmic reticulum (ER) subdomain. In roots, non-ER vesicles were a dominant myosin cargo. Myosin XI-K showed a striking polar localization at the tips of growing, but not mature, root hairs. These results strongly suggest that a major mechanism whereby myosins contribute to plant cell physiology is vesicle transport, and that this activity can be regulated depending on the growth phase of a cell.

## Introduction

**Figure 1:**
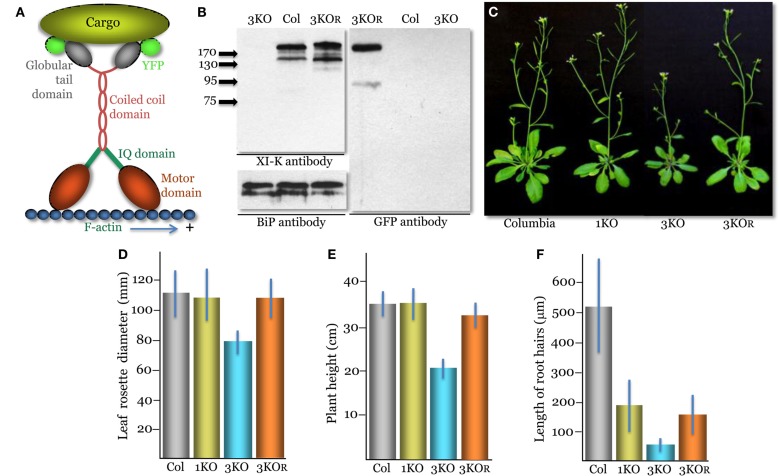
**Phenotypes of the triple myosin knockout plants *xi-k xi-1 xi-2* (3KO) transformed to express XI-K:YFP (designated 3KOR, R – for rescue of the myosin XI-K function)**. **(A)** A diagram showing the domain structure of the YFP-tagged myosin XI-K dimer bound to the F-actin and a cargo. **(B)** Immunoblot analysis of the leaf extracts using the myosin XI-K-specific (left upper panel), BiP-specific (left bottom panel; loading control), or GFP-specific (right panel) antibodies; arrows show protein marker positions and their mol. mass in kDa. Col, the control Columbia-0 plants. **(C)** Representative images of the control Columbia-0, *xi-2* knockout (1KO), 3KO, and 3KOR plants. **(D)** Mean diameter of the leaf rosettes. **(E)** Mean plant heights. **(F)** Mean lengths of the root hairs. **(D–F)** The mean standard deviation for each variant is shown as blue bar.

Myosins are universally conserved, essential molecular motors in eukaryotes (Vale, 2003; Richards and Cavalier-Smith, 2005). The class V/XI myosins of fungi, animals, and plants encompass an actin-binding motor domain, a regulatory IQ domain, a dimerization coiled coil domain, and a cargo-binding globular tail domain (Figure 1A; Trybus, 2008). The functions of myosin V have been investigated extensively in budding yeast, where these motors are required for organelle inheritance and the transport of secretory vesicles (Bretcher, 2003). Myosin interactions with their endomembrane cargoes involve Rab GTPases, as well as organelle-specific receptors (Fagarasanu et al., 2010; Rossi and Brenwald, 2011; Santiago-Tirado et al., 2011).

Although the yeast model continues to provide insight into myosin V function, it cannot account for the complexity of multicellular eukaryotes. Recently, myosin V functions ranging from synaptic plasticity to epithelial polarization to long-range vesicle transport in oocytes were discovered in vertebrate models (Wang et al., 2008; Roland et al., 2011; Schuh, 2011). Vertebrates possess only three myosin V genes, but these give rise to multiple cell type-specific myosin isoforms via alternative splicing (Roland et al., 2009).

Another significant development was the emergence of *Arabidopsis* as a model for investigating myosin functions in complex eukaryotes. *Arabidopsis* possesses 13 myosins XI, which are closely related to the myosin V class. In addition to alternative splicing, *Arabidopsis* myosins exhibit cell type-specific expression patterns (Peremyslov et al., 2011). Phenotypic analysis of myosin XI gene knockouts revealed critical roles of the myosins XI-K, XI-2, and XI-B in polarized elongation of root hairs (Ojangu et al., 2007; Peremyslov et al., 2008, 2010; Prokhnevsky et al., 2008). Another subset of myosins (XI-K, XI-1, XI-2, and XI-I) was implicated in diffuse growth of epidermal and mesophyll cells (Prokhnevsky et al., 2008; Peremyslov et al., 2010). Strikingly, the extent of the myosins’ contributions to this process was proportional to the normal cell size, leading to the proposition that the emergence of larger and more diverse cell types in plant evolution was facilitated by the proliferation of myosins and their associated long-range endomembrane transport activity (Peremyslov et al., 2010).

At the subcellular level, the rapid trafficking of several larger organelles (Golgi stacks, mitochondria, and peroxisomes; Avisar et al., 2008; Peremyslov et al., 2008; Prokhnevsky et al., 2008), as well as the streaming of endoplasmic reticulum (ER; Ueda et al., 2010), relies entirely on the myosins XI (Peremyslov et al., 2010). In addition, the highly expressed myosins XI-K, XI-1, and XI-2 are involved in F-actin organization (Peremyslov et al., 2010; Ueda et al., 2010).

However, it was not clear which of the myosin activities are required for cell growth, and whether the larger organelles are the principal myosin cargoes. To approach these problems, we focused on myosin XI-K, which is a principal player in multiple myosin-dependent processes in *Arabidopsis*. We generated a functional, yellow fluorescent protein (YFP)-tagged XI-K variant (XI-K:YFP), and used it to identify endomembrane vesicles as the principal myosin XI-K cargoes in cells growing in either a diffuse or polarized manner. Together with previous studies, this novel insight suggests that myosins contribute to cell growth via driving vesicular transport toward the expanding plasma membrane and cell wall. Furthermore, it puts an emphasis of future plant myosin research squarely on the identification of the myosin receptors for these transport vesicles.

## Materials and Methods

### YFP-tagged myosin XI-K, *Arabidopsis* transformation, and immunoblotting

A genomic copy of the myosin *XI-K* gene (AT5G20490) tagged by insertion of the YFP ORF (*XI-K:YFP*) was generated as described for the FLAG-tagged XI-K (Peremyslov et al., 2010). The YFP ORF was PCR amplified and inserted in a pMDC32 binary vector carrying a genomic copy of the *XI-K* using overlapping PCR. The resulting plasmid was mobilized into *Agrobacterium tumefaciens* GV3101 and used to transform the 3KO *xi-k xi-1 xi-2* plants (Peremyslov et al., 2010) by floral dipping. The identifiers for the T-DNA insertion lines used to generate 3KO plants were Salk_067972, Salk_019031, and Salk_055785, respectively. Transgenic plants designated 3KOR were selected using Hygromycin-containing medium and YFP imaging. Immunoblotting using a rabbit polyclonal XI-K antibody, rabbit polyclonal antibody to ER binding protein (BiP; a gift from Dr. J. Denecke), or mouse monoclonal GFP antibody (Roche) was done as described (Peremyslov et al., 2008). All four originally selected, independent lines of transformed plants showed very similar levels of XI-K:YFP expression and virtually indistinguishable phenotypes. One of these lines was selected for all following analyses. To compare the expression levels of myosin XI-K in Columbia-0 to that of XI-K:YFP in 3KOR line plants, the immunoblots were quantified by measuring mean band intensity normalized to that of a loading control (BiP; *n* = 4 for each variant). The phenotypes of plant lines presented in Figures 1D–F were characterized as described (Peremyslov et al., 2010); statistical analyses of the data, including standard deviations shown as error bars and *t*-tests, were done using Microsoft Excel package. The *p* values, corresponding to pairwise comparisons of data sets, are presented in the text under Results.

To generate transgenic lines expressing compartment-specific markers, a binary vector pCB301 was modified to accommodate an *UBQ10* promoter (Geldner et al., 2009) and a polyadenylation signal, and used to generate N-terminal fusions of the mTurquoise fluorescent protein (Goedhart et al., 2010) with the actin-binding domain LifeAct (Goedhart et al., 2010), the transmembrane domain of *N*-acetylglucosaminyl transferase I (NAG; Grebe et al., 2003), or SCAMP2 (Toyooka et al., 2009). An ER-targeted CFP was described before (Peremyslov et al., 2010). The marker-expressing 3KOR plants were selected using glufosinate. The transgenic plant lines generated in Columbia or 3KOR genetic background had normal developmental phenotypes under optimal growth conditions. This was also the case for the LifeAct-mTurquoise plants in accord with recent independent work (van der Honing et al., 2011).

### Confocal laser scanning microscopy

The microscopy was done using a Zeiss LSM 510 microscope and a 63 × 1.4 NA objective. Plants expressing myosin XI-K:YFP and mTurquoise- or CFP-tagged markers were grown on vertical plates and used for imaging the lower midrib epidermal cells or root hairs. For YFP, the Argon laser was used with a 514 nm dichroic beam splitter and a 530–600 nm bandpass filter. To reliably avoid bleed-through of the yellow and blue fluorophore signals, dual imaging of YFP and mTurquoise (or CFP) was done using the single track and a 405 nm Diode laser at 1% transmission power and Argon laser at 10% transmission power, respectively. We also used a HFT 405/514 nm beam splitter and a 420–480 nm bandpass filter for mTurquoise, and a 530–600 nm bandpass filter for YFP.

To reconfirm that no bleed-through has occurred, a series of control scans for each sample was carried out. In these scans, either of two lasers was turned off during the scan. The disappearance of the signal in the corresponding channel while the image in the alternative channel remained visible proved that the bleed-through between channels was below the detectable level.

Co-localization studies were done using the JACoP plugin for ImageJ software (Bolte and Cordelieres, 2006). Prior to performing calculations, background correction was done using a set of built-in tools available in the ImageJ. First, merged color dual-channel images were separated into individual channels using the “Color split” feature, and Median filter (radius 2 pixels) was applied to each channel independently. Then, the background subtraction was done to remove residual background by using the “Rolling ball” tool (radius 20 pixels). Threshold within the JACoP plugin was set by using automatically calculated values suggested by the program to avoid user bias. Both Pearson’s coefficient and Manders coefficients M1 and M2 were calculated and compared. Each coefficient shown in Table S1 in Supplementary Material represents a mean and standard deviation for 20 images derived from five plants. The Pearson’s coefficient is commonly used to determine extent of overlap between image pairs via analyzing similarity between shapes while ignoring the signal intensities. A value of 1.0 represents perfect correlation and a value 0.0 represents no correlation (Manders et al., 1993). The Manders’ M1 and M2 coefficients are the percentage of pixels in one channel that overlaps with the signal in other channel (Bolte and Cordelieres, 2006).

For the fluorescence recovery after photobleaching (FRAP) analysis of plants expressing myosin XI-K:YFP or SCAMP2-YFP, all Argon laser lines (458, 477, 488, and 514 nm) and Diode 405 nm laser line were used simultaneously at 100% transmittance for 50 iterations at maximum scanning speed. The bleaching routine started with two pre-bleach scans followed by the bleaching scan. After bleaching, images were taken at the 8% Argon laser transmission for 60 s. Fluorescence recovery was measured using the LSM 510 software (ROI Mean plugin). Fluorescence intensities in the unbleached regions were measured to ensure that no substantial fluorescence reduction occurred during the observation period. The FRAP measurements for each experimental variant were done using 10 distinct root hairs; mean and standard deviation values were calculated for each time point as shown in Figures 3H,I.

### Subcellular distribution of myosin XI-K

Membrane association assays were done using young Columbia-0 leaves. The 1,000 *g* supernatant was prepared, and equal fractions were supplemented with either homogenization buffer or 1% Triton X-100. Aliquots were centrifuged at 100,000 *g*, and the resulting pellets were adjusted to the same volume as supernatants, and analyzed by immunoblotting.

Membrane fractionation of leaf tissue was done essentially as described by Zhang et al. (2010); root fractionation as described by Preuss et al. (2004). Fractions were analyzed by immunoblotting using the following antisera to known organelle markers: rabbit polyclonal to ER BiP; rabbit polyclonal to Golgi marker SecP21 (Agrisera, Sweden); mouse monoclonal anti-GFP antibody to detect NAG-YFP and EYFP-RabA4b (Roche); rabbit polyclonal to RabA4b (a gift from Dr. E. Nielsen); rabbit polyclonal to pre-vacuolar compartment (PVC) marker Syp21 (a gift from Dr. N.V. Raikhel); rabbit polyclonal to the plasma membrane marker H^+^ATPase (Agrisera, Sweden); and mouse polyclonal antibodies to the exocyst subunit Sec6 (gift from Dr. V. Zarsky). The fractionation experiments were repeated at least three times for leaf and root extracts each, producing consistent fractionation profiles represented in Figure 4.

## Results

### Generation of the fluorophore-tagged myosin XI-K

*Arabidopsis* myosin XI-K is encoded by a large gene transcript that undergoes alternative splicing (Peremyslov et al., 2011). To ensure proper gene expression and regulation, we placed a *YFP* tag at the 3′ end of the coding sequence in a genomic *XI-K* clone to produce XI-K:YFP (Figure 1A).

Although XI-K is the most significant contributor to myosin-dependent cell growth and organelle transport, two paralogous myosins, XI-1 and XI-2, have partially redundant functions in these processes (Peremyslov et al., 2010). Therefore, to determine if XI-K:YFP is functionally competent, we transformed the modified genomic clone into *xi-k xi-1 xi-2* triple knockout (3KO) plants. The 3KO plants exhibit reduced size and possess much shorter root hairs compared to the wild type (Peremyslov et al., 2010).

Immunoblot analyses confirmed expression of the ∼200 kDa XI-K:YFP product in the transformed *xi-k xi-1 xi-2 XI-K:YFP* plants (Figure 1B). The expression level of XI-K:YFP was not significantly different from that of the wild type XI-K in the Columbia-0 control (110 ± 7%; *p* = 0.2). Phenotypic characterization of these plants showed that their mean rosette diameter and height were significantly higher than those of 3KO plants (*p* < 1 × 10^−11^; Figures 1C–E). Furthermore, the rosette diameter of these plants was not significantly distinct from that of the Columbia control plants (*p* = 0.44). Although marginally significant (*p* = 0.001), the height of transgenic plants was only 8% lower than that of Columbia compared to ∼40% reduction seen in 3KO plants (Figure 1E). These results are in agreement with accessory roles for myosins XI-1 and XI-2 in plant growth, which cause no defects when inactivated on their own (Prokhnevsky et al., 2008; Peremyslov et al., 2010; e.g., the *xi-2* 1KO plants in Figures 1C–E).

Characterization of root hairs also confirmed the functional competence of XI-K:YFP. Myosins XI-K and XI-2 provide similar, additive contributions to root hair elongation: inactivation of either gene results in approximately threefold root hair length reduction (Peremyslov et al., 2008; Prokhnevsky et al., 2008). Accordingly, expression of XI-K:YFP resulted in a approximately threefold increase in the root hair length (*p* < 1 × 10^−30^) compared to that of 3KO plants, similar to the length of the *xi-2* 1KO root hairs (Figure 1F). We concluded that the ability of XI-K:YFP to rescue the plant and root hair growth defects in the 3KO mutant validated the functional activity of this myosin. For brevity, the *xi-k xi-1 xi-2 XI-K:YFP* plant line was dubbed 3KOR – R for rescued.

### Localization patterns of the myosin XI-K:YFP in leaf cells

**Figure 2:**
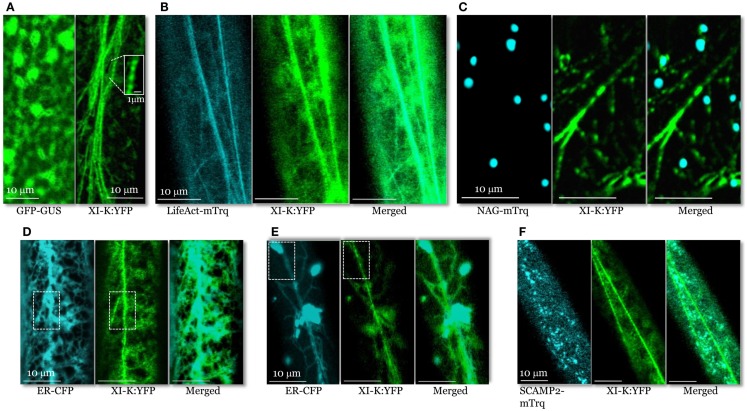
**Confocal images of the leaf midvein epidermal cells in 3KOR plants expressing XI-K:YFP and different compartment- specific markers**. **(A)** In contrast to cytosolic GFP-GUS (left panel), XI-K:YFP (right panel) is associated with vesicle-like bodies shown in the boxed close-up inset. **(B)** Co-localization of XI-K:YFP and F-actin marker LifeAct-mTurquoise. **(C)** Co-localization of XI-K:YFP and Golgi marker NAG-mTurquoise. **(D,E)** Co-localization of XI-K:YFP and the ER-CFP marker in the leaf **(D)** and root **(E)** epidermis. **(F)** Co-localization of XI-K:YFP and secretory vesicle marker SCAMP2-mTurquoise.

The elongated epidermal cells of *Arabidopsis* leaf midrib are a favored model for myosin research due to their imaging accessibility and extensive, myosin-dependent trafficking of organelles. Observation of these cells in the 3KOR plants using confocal laser scanning microscopy revealed a striking pattern of XI-K:YFP distribution: most of the fluorescence was associated with motile, vesicle-like bodies moving along linear tracks (Figure 2A; Movie S1 in Supplementary Material). The remaining fluorescent material appeared in scattered patches. This distribution pattern suggested that most of the myosin XI-K:YFP pool is associated with endomembrane vesicles, whereas a relatively small fraction of myosin was present elsewhere in cytosol.

To determine if XI-K:YFP co-localizes with F-actin, the 3KOR plants were transformed to express microfilament marker LifeAct-mTurquoise (Era et al., 2009; Vidali et al., 2009; Goedhart et al., 2010). XI-K:YFP and LifeAct-mTurquoise were largely co-localized (Figure 2B), with a relatively high mean Pearson co-localization coefficient of 0.8, as well as a high degree of mutual overlap between the pools of XI-K:YFP and LifeAct-mTurquoise (mean Manders coefficients of 0.65 and 0.76; Table S1 in Supplementary Material).

An analogous co-localization analysis of XI-K:YFP and the Golgi marker NAG-mTurquoise showed that only a small fraction of the myosin pool was associated with Golgi stacks (Figure 2C; Pearson coefficient of 0.13; Table S1 in Supplementary Material). Furthermore, the fraction of XI-K:YFP overlapping Golgi was only 0.08, as defined by Manders coefficient (Table S1 in Supplementary Material). These data indicate that Golgi stacks are not the primary cargo of myosin XI-K. It should also be emphasized that, as seen in the merged image (Figure 2C), the vesicle-like bodies tagged with XI-K:YFP are smaller and much more numerous compared to NAG-mTurquoise-tagged Golgi stacks.

Analysis of XI-K:YFP co-localization with the ER-CFP marker (Peremyslov et al., 2010) revealed a dramatically different picture (Figure 2D) of an extensive overlap (Pearson coefficient of 0.75; Table S1 in Supplementary Material). Interestingly, although most of the myosin XI-K:YFP co-localized with ER (Manders coefficient of 0.90), a smaller fraction of ER overlapped with myosin (0.37; Table S1 in Supplementary Material). This distribution pattern suggested that only a motile subdomain of ER was associated with myosin, whereas most of ER is myosin-free (Figure 2D).

It should be pointed out, however, that the organization of the myosin XI-K:YFP-decorated material was distinct from that of ER even in areas of maximal overlap (boxed areas in Figure 2D). Whereas the myosin-associated particles had a “beads-on-a-string” appearance, ER exhibited a characteristic network of more flat cisternae. This difference was even more pronounced in root epidermal cells (Figure 2E). In these cells, a large fraction of ER was contained in spindle-shaped ER bodies (Yamada et al., 2008) interconnected by thin strands. As clearly seen in Figure 2E, the bright ER bodies scarcely if at all overlapped with myosin-associated material that again, appeared as beads. These data suggest that only a fraction of the ER is interacting with myosin. One possibility is that this fraction is composed of motile, ER-derived vesicles.

To further explore the nature of the myosin-associated material, we investigated XI-K:YFP co-localization with vesicles labeled by the secretory carrier membrane protein 2 (SCAMP2), a marker of post-Golgi vesicle clusters observable by confocal microscopy (Toyooka et al., 2009). To this end, we generated a SCAMP2 marker fused with mTurquoise (SCAMP2-mTurquoise; Figure 2F). Our analysis showed a substantial level of co-localization (Pearson coefficient of 0.62), but relatively little overlap of XI-K:YFP with SCAMP2-mTurquoise (0.15; Table S1 in Supplementary Material) indicating that SCAMP2-labeled vesicles represent only a fraction of the myosin XI-K cargoes in this cell type. It should be accentuated that the SCAMP2-specific secretory vesicle clusters were comparable in size and appearance to myosin XI-K:YFP-tagged vesicular bodies (Figure 2F).

Previous research demonstrated the myosin XI-dependent nature of the Golgi and ER transport (Peremyslov et al., 2010; Ueda et al., 2010). In contrast, the first experimental indication of the direct myosin XI involvement in the transport of secretory vesicles was provided only recently (Amari et al., 2011). To determine if the limited co-localization of XI-K:YFP and SCAMP2-containing vesicles is functionally relevant, we compared SCAMP2-YFP motility in Columbia and 3KO plants. As seen in Movies S2 and S3 in Supplementary Material, myosin inactivation resulted in dramatic reduction of the long-distance movement of marker-tagged vesicles, indicating that their transport is myosin-dependent.

Taken together, these co-localization and genetic analyses strongly suggested that endomembrane vesicles rather than larger organelles such as Golgi are the principal myosin cargoes. Because the average diameter of the typical individual vesicles is below the resolution of a light microscope, we assume that the myosin- or SCAMP2-labeled structures seen in Figure 2 are vesicle clusters, in accord with a common agreement in this filed. Furthermore, the average size and pattern of the myosin XI-K:YFP-associated vesicular clusters are dramatically distinct from those of the larger organelles such as Golgi (cf. Figures 2A,C), mitochondria, or peroxisomes. These organelles do not exhibit a “beads-on-a-string” appearance characteristic of motile, myosin-containing vesicular clusters. At ∼0.5 μm, these roughly isometric clusters are much smaller than, e.g., typical elongated mitochondria (2–5 μm) present in *Arabidopsis* leaf cells. It looks likely that the myosin transport-competent vesicles include ER-derived, secretory, and possibly other vesicle types.

### Polarized myosin localization in growing root hairs

**Figure 3:**
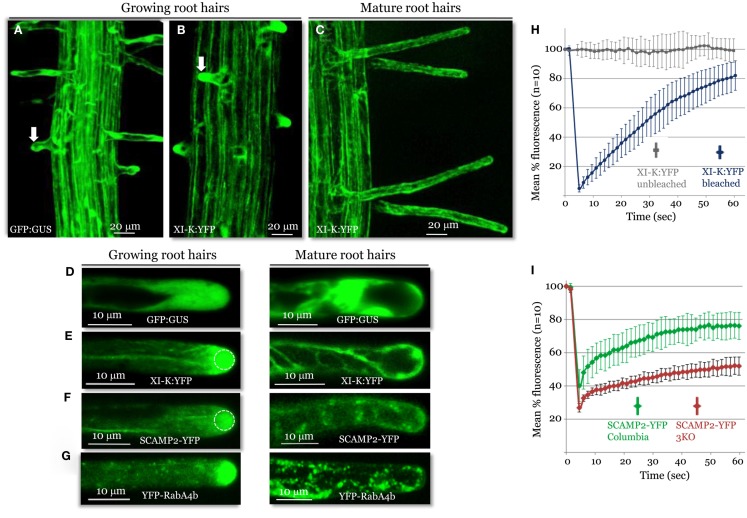
**Confocal imaging analysis of XI-K:YFP and vesicle-specific, YFP-tagged markers in growing and mature root hairs**. Localization patterns of GFP:GUS control **(A,D)**, XI-K:YFP **(B,C,E)**, SCAMP2:YFP **(F)**, and YFP-RabA4b **(G)** are shown. Arrows in **(A,B)** mark similar root hairs showing myosin XI-K:YFP accumulation in the cell tip **(B)** not observed in **(A)**. **(H)** Fluorescence recovery after photobleaching (FRAP) analysis of the growing root hair tips expressing XI-K:YFP. **(I)** FRAP analysis of growing root hairs in Columbia and 3KO plants (*n* = 10 for each variant), stably transformed to express SCAMP2-YFP. Identical photobleaching settings were used for all trials. Dashed circles in **(E,F)** indicate the photobleached areas in FRAP experiments shown in **(H,I)**.

A striking pattern of myosin XI-K:YFP localization was revealed in root hairs, which grow via a polarized elongation mechanism (Cole and Fowler, 2006; Tominaga-Wada et al., 2011). A much higher level of this myosin was present in the growing root hairs, compared to the cell base from which the root hairs extend (Figure 3A). Within the elongating hairs, XI-K:YFP was dramatically enriched in the tip area, whereas outside the tips, XI-K:YFP was associated with vesicles moving along F-actin bundles (Figure 3C, left panel). It should be emphasized that the XI-K:YFP-labeled structures in the tip showed highly dynamic behavior suggestive of extensive vesicular trafficking (Movie S4 in Supplementary Material). This behavior is illustrated by the rapid FRAP of myosin XI-K:YFP in root hair tips (Figure 3F), which indicates that the myosin pool in the tip is constantly renewing rather than forming a resident population. In contrast, there was no preferential myosin accumulation at the tips of the mature root hairs (Figures 3B,C, right panel). The myosin-labeled structures in these cells exhibited a relatively steady flow throughout the cell (Movie S5 in Supplementary Material).

The observed growth stage-dependent myosin localization in root hairs was very similar to that of the secretory vesicle markers SCAMP2-YFP (Toyooka et al., 2009) and YFP-RabA4b (Preuss et al., 2004; Figures 3D,E), which accumulated at the tips of growing, but not mature, root hairs. The role of the myosins in vesicular trafficking was further addressed using 3KO plants expressing the SCAMP2-YFP reporter. Inactivation of the three myosins results in a substantial reduction in the dynamics of the SCAMP2-YFP-labeled vesicles. Instead of the rapid processive transport seen in the control, these vesicles showed slow, zigzag-like movement in the 3KO root hairs (Movies S6 and S7 in Supplementary Material). This effect was validated by FRAP analysis, which demonstrated slower fluorescence recovery in the 3KO mutant compared to control plants (Figure 3G).

Collectively, these data suggest that myosin XI-K is involved in long-distance transport of vesicles along F-actin bundles, and possibly in short-distance delivery to the plasma membrane of vesicles amassed at the tip of a growing root hair. In addition, the pattern and dynamics of myosin XI-K localization in root hairs shows striking similarity to those reveled in moss protonemal cells that also grow via polarized elongation (Vidali et al., 2010).

### Myosin co-fractionation with endomembranes

**Figure 4:**
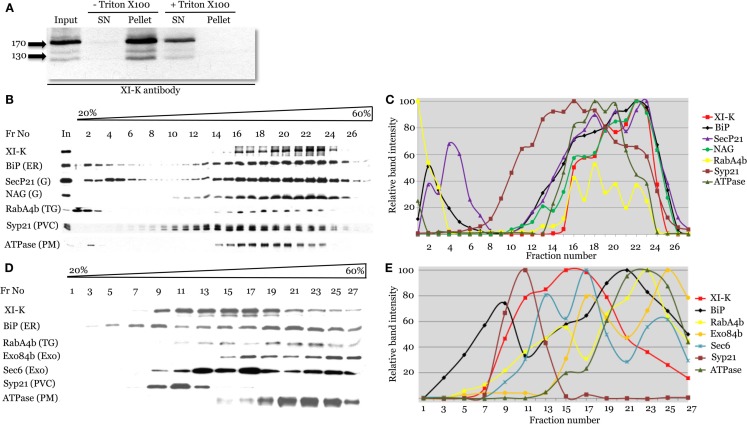
**Cell fractionation analysis of the distribution of myosin XI-K and subcellular compartment markers using immunoblotting**. **(A)** Analysis of the *Arabidopsis* leaf extracts shows presence of the myosin XI-K in the microsomal pellet; treatment by Triton X-100 results in XI-K release to detergent-soluble supernatant (SN). Arrows show protein marker positions and their mol. mass in kDa. **(B,C)** Fractionation of the leaf extracts in the 20–60% sucrose gradients. **(B)** Immunoblot analyses of the gradient fractions (fraction numbers or Fr No are shown at the top; In, input) using XI-K or cell compartment-specific antibodies as indicated at the left. G, Golgi; TG, trans-Golgi; PVC, pre-vacuolar compartment; PM, plasma membrane. **(C)** Quantification of the protein band intensity for the immunoblot analyses shown in **(B)**. **(D,E)** Fractionation of the root extracts in the 20–60% sucrose gradients similar to the analysis shown in **(B,C)**. Exo, exocyst components; other designations are as in **(B)**.

To address the physical association of myosin XI-K with endomembranes supported by imaging, we used leaf extract fractionation and an XI-K-specific antibody (Peremyslov et al., 2008). Clarified leaf extracts from Columbia plants were centrifuged to separate the soluble cytosolic fraction and the microsomal fraction. As seen in Figure 4A, virtually all (∼96%) of the myosin was present in the microsomal pellet. In contrast, when the extract was treated with the Triton X-100 to solubilize the membranes, 99% of the myosin was found in the supernatant. This result is fully compatible with endomembranes being the major myosin XI-K cargo.

To address the nature of the endomembranes bound by myosin XI-K, the extracts were fractionated in an isopycnic sucrose gradient, and individual fractions were analyzed by immunoblotting, using antibody markers specific for distinct endomembrane compartments. In these fractions, the XI-K peak was well separated from those of the Syp21 and H^+^ATPase markers for PVC (da Silva Conceicao et al., 1997) and plasma membrane, respectively (Figures 4B,C). On the other hand, the myosin peak corresponded well to the peaks of the ER marker BiP, and Golgi markers SecP21 and NAG. The distribution of the trans-Golgi/secretory vesicle marker RabA4b (Preuss et al., 2004) also corresponded broadly to that of the myosin (Figures 4B,C). These results suggested that most of the myosin XI-K in leaf cells is associated with the ER-, organelle-, and secretory vesicles-derived membranes.

We also investigated the myosin fractionation pattern in extracts from Columbia roots. Interestingly, with root as the source, myosin XI-K clearly separated not only from the PVC and plasma membrane, but also from the ER (Figures 4D,E). The best correspondence was observed between the myosin peak and that of exocyst protein Sec6 (Hála et al., 2008), likely to be associated with secretory vesicles. The myosin peak was also coincident with one of the peaks for the vesicular marker RabA4b (Figures 4D,E). We concluded that in the root cells, most of the myosin XI-K is bound not to the ER, but rather to the secretory vesicles.

Thus, fractionation results have validated myosin association with endomembranes, and were consistent with imaging results suggesting vesicular cargo. The fractionation patterns of myosin and endomembrane compartment-specific markers indicated that major myosin cargoes could vary between two different tissues.

## Discussion

The discovery that myosins XI contribute to the growth of plant cells in both *Arabidopsis* and moss initiated a quest for an underlying mechanism (Peremyslov et al., 2010; Vidali et al., 2010). Here we propose that this mechanism is based on myosin-driven traffic through the secretory pathway to the plasma membrane, and provide first experimental support for this model. We also posit that vesicle transport is required for homeostasis in mature cells.

It stands to reason that the bulk of the cellular myosin “motor pool” is engaged in transporting cargoes that are most important for cell growth and function, but defining such cargoes has been an elusive goal. Since 2001, studies have reported, in addition to dispersal in the cytosol, myosin XI association with mitochondria, plastids, peroxisomes, ER, Golgi, nuclear envelope, plasma membrane, unidentified organelles, and vesicles (Liu et al., 2001; Hashimoto et al., 2005; Li and Nebenfuhr, 2007; Sparkes et al., 2008; Sattarzadeh et al., 2011). The disparate conclusions drawn from these studies are difficult to reconcile; perhaps, the only semblance of consensus in this field was apparent association of myosins with larger organelles such as mitochondria, peroxisomes, and Golgi.

In contrast, this study demonstrated the predominant association of a functional myosin XI-K:YFP with endomembrane vesicles transported along F-actin bundles. The first line of evidence was provided by imaging, which showed XI-K:YFP primarily decorating vesicular bodies rather than larger organelles (Figure 2A). Quantitative analysis confirmed that less than 7% of the myosin XI-K:YFP was associated with the Golgi (Table S1 in Supplementary Material).

Because ∼90% of the XI-K:YFP co-localized with a subset of the ER (Figure 2D; Table S1 in Supplementary Material), it could be proposed that most of this myosin is engaged in driving ER flow. However, ER is not necessarily the principal myosin XI-K cargo. The concentration of both ER membranes and myosin along F-actin bundles (Figures 2A,B,D) may imply either true physical interaction, or independent association of each with F-actin. The fine structure of the ER sheets and strands compared to XI-K:YFP-decorated bead-like structures (Figures 2D,E) appears to support the latter possibility.

A striking pattern of myosin localization was revealed in growing root hairs, where the bulk of XI-K:YFP was localized to the cell tip (Figures 3A,C), from which F-actin bundles are excluded (Baluska et al., 2000; Peremyslov et al., 2010). This polarized localization was similar to that of the secretory vesicle markers SCAMP2 and RabA4b, consistent with extensive, myosin-driven vesicle delivery to the tip. The polarization of the myosin and vesicular markers was lost in mature root hairs (Figures 3C–E).

To determine the role of myosins XI in the trafficking of SCAMP2-YFP-tagged secretory vesicles, we assessed marker dynamics in root hairs of the 3KO mutant in which myosin XI-K and two other highly expressed myosins were inactivated. Instead of the typical rapid transport, shorter mutant hairs exhibited both reduced vesicle movement and slower recovery of fluorescent signal at the tip following photobleaching (Figure 3G; Movies S6 and S7 in Supplementary Material). These data support a role for myosin XI-K in driving the long-distance vesicle transport toward the tip, and potentially the short-distance transport of vesicles to the plasma membrane. In addition, these data provide a second line of evidence for vesicles being a myosin XI-K cargo. It should be noted, however, that our data do not distinguish between a direct or indirect role for myosin in the transport of SCAMP2-YFP-tagged vesicles. It is equally feasible that these vesicles are directly bound to myosin XI-K, or that their transport occurs via cytosolic flow generated from direct myosin transport of an alternative vesicle type.

Interestingly, moss myosin XI is also concentrated in the tips of growing protonemal cells as shown in an elegant recent work by Vidali et al. (2010). The evolutionary roots of a role for myosin in polarized growth appear to be very deep, given myosin V involvement in polarization of budding yeast cells and the ancient origin of the myosin V/XI class (Bretcher, 2003; Richards and Cavalier-Smith, 2005).

The third line of evidence compatible with vesicle transport as the primary myosin XI-K function comes from membrane fractionation analysis. Unexpectedly, virtually all of the myosin XI-K was associated with membranes (Figure 4A). Therefore, most of this myosin is in active, cargo-bound form, making an important distinction with vertebrate myosin Va, which adopts an inactive conformation upon cargo detachment (Krementsov et al., 2004). A tight engagement of myosin with the endomembranes may in part explain the more extensive cytoplasmic dynamics in plant cells compared to that in other eukaryotes.

Perhaps the most pressing question posed by this study is the identity of the vesicles transported by myosin. On the one hand, the high degree of the overlap between XI-K:YFP and the ER suggests that the myosin-decorated vesicles are ER-derived. On the other hand, this result does not exclude myosin association with post-Golgi secretory vesicles, given that the distributions of the vesicle markers YFP-RabA4b and SCAMP2 overlap with that of the myosin XI-K. Furthermore, fractionation of root extracts showed clear separation between ER and myosin peaks (Figures 4D,E) and correspondence of the myosin peak with those of exocyst component, likely to be associated with exocytic vesicles (Hála et al., 2008).

Two working models could be proposed to account for myosin-driven vesicle trafficking in plant cells. One model posits that vesicles are mobilized via interaction between vesicle type-specific myosin receptors and cognate myosins XI. Among the strongest candidate receptors are Rabs, which interact with myosins V in yeast and vertebrates, and are thought to be important determinants of vesicle type identity (Woollard and Moore, 2008). The 57 Rabs encoded by the *Arabidopsis* genome provide a vast potential resource for regulation of myosin-dependent vesicle transport. It is important to emphasize that although transport of SCAMP2 secretory vesicles requires myosin, only a fraction of these vesicles co-localizes with myosin. The most parsimonious hypothesis explaining these data posits that the myosin-associated vesicle pool is composed of multiple types of vesicles. In other words, a novel class of motile vesicles, comprised of subpopulations of vesicle types defined by vesicle-specific markers (such as Rabs or SCAMP2), appears to be a distinct possibility. A critical proof of this hypothesis could be provided by identification of the vesicular receptors responsible for direct myosin binding, therefore defining this novel class of vesicles.

An alternative model proposes that only one or a few vesicle types are the *bona fide* myosin cargoes, whereas other vesicles and organelles move passively, with cytosolic flow. Distinguishing between these models will also require identification of the myosin receptors, as well as the primary myosin cargoes. Correspondingly, characterization of myosin receptors, as well as the composition of the associated motile, myosin-decorated vesicles, will be a current focus of research in the plant myosin field.

Another question prompted by this work are the discrepancies between this and previous studies regarding myosin localization. Several of these studies used immunocytochemical approach and antibodies of variable, not always well defined specificity (Liu et al., 2001; Wang and Pesacreta, 2004; Hashimoto et al., 2005; Romagnoli et al., 2007; Yokota et al., 2009). The outcomes of corresponding analyses varied from cytosolic co-localization with molecular chaperone TCP-1α to mitochondria to peroxisomes to ER, and are often mutually exclusive. On the other hand, many of the recent studies involved transient expression of the various, fluorophore-tagged myosin fragments rather than full-length proteins (Li and Nebenfuhr, 2007; Reisen and Hanson, 2007; Sparkes et al., 2008; Avisar et al., 2009, 2012; Natesan et al., 2009; Sattarzadeh et al., 2009, 2011). Even within the same experiment, this approach could result in distinct localization patterns among different cells, likely due to variation in protein accumulation levels (Li and Nebenfuhr, 2007; Sattarzadeh et al., 2011). Even more alarmingly, localization patterns of these myosin fragments do not correspond to functional effects caused by their expression (Avisar et al., 2012). These patterns were further affected by the domain structure of the expressed myosin fragments, which may in turn determine the protein’s ability to interact with appropriate cargoes. Because our work uses a full-length, functional myosin whose expression is driven by its native regulatory elements, the authenticity of the resulting localization pattern is much more certain. This notion is further supported by very similar localization patterns described for a full-size, functional, fluorophore-tagged myosin XI in *Arabidopsis* root hairs (this work) and protonemal moss cells that share the polar cell growth mechanism (Vidali et al., 2010).

In conclusion, we propose that the principal function of myosin XI-K is trafficking of vesicles through the endomembrane system, including delivery of secretory vesicles to areas of cell growth. We also suggest that the myosin XI-K-associated vesicles encompass subpopulations of the distinct types of vesicles mobilized via attachment of myosins to cognate vesicular receptors. This model is in accord with the data presented herein, and with previous genetic analysis demonstrating arrest of polarized root hair elongation and reduction in the diffuse cell growth in myosin-deficient plants (Peremyslov et al., 2010). In mature cells, myosin-driven vesicle transport could be required for cell homeostasis, e.g., delivery of plasma membrane proteins. In agreement with this, myosins XI were recently implicated in the steady-state transport of certain integral membrane proteins (Amari et al., 2011). The approach described here for the myosin XI-K provides a clear path to investigating localization patterns and functions of the other flowering plant myosins.

## Conflict of Interest Statement

The authors declare that the research was conducted in the absence of any commercial or financial relationships that could be construed as a potential conflict of interest.

## Supplementary material

Supplementary Table S1 **Co-localization of the myosin XI-K:YFP with the fluorophore- labeled F-actin (LifeAct-mTrq), Golgi (NAG-mTrq), ER (ER-CFP), and secretory vesicles (SCAMP2-Trq)**. Analyses were done in midrib vascular epidermis cells and root epidermis cells. Mean values are given for each coefficient.

Supplementary Movie S1 **Motility of the XI-K:YFP-associated, vesicle-like bodies in the leaf midvein epidermis of *Arabidopsis* xi-k xi-1 xi-2 XI-K:YFP (3KOR) plants**.

Supplementary Movie S2 **Motility of the SCAMP2-containing secretory vesicles in the leaf midvein epidermis of *Arabidopsis* Columbia-0 plants transformed to express SCAMP2-YFP reporter**.

Supplementary Movie S3 **Motility of the SCAMP2-containing secretory vesicles in the leaf midvein epidermis of xi-k xi-1 xi-2 3KO plants transformed to express SCAMP2-YFP reporter**.

Supplementary Movie S4 **Motility of the XI-K:YFP-associated, vesicle-like bodies in the growing root hair of the xi-k xi-1 xi-2 XI-K:YFP (3KOR) plants**.

Supplementary Movie S5 **Motility of the XI-K:YFP-associated, vesicle-like bodies in the mature root hair of the xi-k xi-1 xi-2 XI-K:YFP (3KOR) plants**.

Supplementary Movie S6 **Motility of the SCAMP2-containing secretory vesicles in the growing root hair of *Arabidopsis* Columbia-0 plants transformed to express SCAMP2-YFP reporter**.

Supplementary Movie S7 **Motility of the SCAMP2-containing secretory vesicles in the growing root hair of xi-k xi-1 xi-2 3KO plants transformed to express SCAMP2-YFP reporter**.
