# Supplementary material for: Arabidopsis Myosin XI-K Localizes to the Motile Endomembrane Vesicles Associated with F-actin
Source: Front Plant Sci. 2012 Sep 3;3:184. doi: 10.3389/fpls.2012.00184 (PMC3432474; doi:10.3389/fpls.2012.00184)
Supplement: Supplementary Table S1 — Co-localization of the myosin XI-K:YFP with the fluorophore- labeled F-actin (LifeAct-mTrq), Golgi (NAG-mTrq), ER (ER-CFP), and secretory vesicles (SCAMP2-Trq). Analyses were done in midrib vascular epidermis cells and root epidermis cells. Mean values are given for each coefficient. [file 31774_Dolja_DataSheet1.PDF]

Supplemental table 1

Co-localization of the myosin XI-K:YFP with the fluorophore-labeled F-actin (LifeAct-mTrq), Golgi (NAG-mTrq), ER (ER-CFP), and secretory vesicles (SCAMP2-Trq). Analyses were done in midrib vascular epidermis cells and root epidermis cells. Mean values are given for each coefficient

	Pearson coefficient	Manders coefficients	
		Fraction of A* overlapping XI-K:YFP	Fraction of XI-K:YFP overlapping A
F-actin	0.803 ± 0.056	0.764 ± 0.073	0.645 ± 0.106
Golgi	0.128 ± 0.065	0.342 ± 0.108	0.078 ± 0.033
ER	0.752 ± 0.142	0.369 ± 0.132	0.903 ± 0.100
ER-root	0.641 ± 0.120	0.507 ± 0.154	0.769 ± 0.122
Secretory vesicles	0.620 ± 0.094	0.467 ± 0.187	0.152 ± 0.088

A* corresponds to subcellular domains listed in the left-most column
